# Supplementary material for: Evolving scattering networks for engineering disorder
Source: Nat Comput Sci. 2023 Feb 13;3(2):128–38. doi: 10.1038/s43588-022-00395-x (PMC10766560; doi:10.1038/s43588-022-00395-x)
Supplement: Supplementary file 1 — Supplementary Notes 1–14, Figs. 1–14 and Algorithm 1. [file 43588_2022_395_MOESM1_ESM.pdf]

nature computational science

Article https://doi.org/10.1038/s43588-022-00395-x

Evolving scattering networks for engineering disorder

In the format provided by the authors and unedited

Supplementary Information for "Evolving scattering networks for engineering disorder"

Supplementary Note 1. Scattering and structure factor

Supplementary Note 2. Classification of impulse scattering responses

Supplementary Note 3. Evolution model of scattering networks

Supplementary Note 4. Generalization to inhomogeneous materials

Supplementary Note 5. Monte Carlo methods for real and reciprocal spaces

Supplementary Note 6. Evolution process using position as a resource

Supplementary Note 7. Performance analysis of the evolution process

Supplementary Note 8. Perturbed structure factor from random defects

Supplementary Note 9. Non-preferential attachment for suppressed scattering

Supplementary Note 10. Degree distributions in the crystals of different *n*

Supplementary Note 11. Evolution of scattering in Poisson and evolving SHU materials

Supplementary Note 12. Extended data and discussion for evolving material screening

Supplementary Note 13. Evolution and network architecture with preferential attachment

Supplementary Note 14. Full-wave calculation for evolving scattering networks

Supplementary Algorithm 1. Pseudo-code for evolution processes

Supplementary Note 1. Scattering and structure factor

For the complete description of wave scattering from a material described by a scattering network, we review the derivation of the structure factor $S_n(\mathbf{k})$ in scattering theory[1]. Consider an incident planewave $\psi_{\text{in}}(\mathbf{r}) = \psi_I \exp(i\mathbf{k}_I \cdot \mathbf{r})$ impinging on the jth point particle at $\mathbf{r}_j$ (Supplementary Fig. 1). When we employ the first-order Born approximation in the Lippmann-Schwinger equation[2], the scattering from the particle is the impulse response excited by the point source from an incident planewave $\psi_{\text{in}}(\mathbf{r})$. The scattering field measured at $\mathbf{R}$ is:

$$\psi_{\text{sc-}j}(\mathbf{R}) = f(\mathbf{k}_S - \mathbf{k}_I) G(\mathbf{R}, \mathbf{r}_j; \mathbf{k}_S) \psi_{\text{in}}(\mathbf{r}_j), \tag{S1}$$

where $\mathbf{k}_S$ is the scattering wavevector depicting $\mathbf{R}$ from $\mathbf{r}_j$, $f(\mathbf{k}_S - \mathbf{k}_I)$ is the single-particle scattering form factor, and $G(\mathbf{R}, \mathbf{r}_j; \mathbf{k}_S)$ is the $\mathbf{k}_S$ component of the Green's function for outgoing waves. For the far-field measurement that allows the asymptotic form of the Hankel function $H_0^{(1)}(z) \sim (2/\pi z)^{1/2}\exp[i(z - \pi/4)]$, the Green's functions for d-dimensional ($d = 1, 2$, and 3) systems have the following form[3]:

$$G(\mathbf{R}, \mathbf{r}_j; \mathbf{k}) = \left(\frac{2\pi}{|\mathbf{k}|}\right)^{\frac{3-d}{2}} e^{i\frac{3-d}{4}\pi} \frac{e^{i\mathbf{k}\cdot(\mathbf{R}-\mathbf{r}_j)}}{|\mathbf{R}-\mathbf{r}_j|^{\frac{d-1}{2}}}. \tag{S2}$$

when $\mathbf{k}$ and $\mathbf{R} - \mathbf{r}_j$ indicate the same direction. From Eqs (S1,S2), the scattering field from an n-particle composite material measured at $\mathbf{R}$ is

$$\psi_{\text{sc}}{}^{n}(\mathbf{R}) = \sum_{j=1}^{n} \psi_{\text{sc-}j}(\mathbf{R}) = \psi_I f(\mathbf{k}_S - \mathbf{k}_I) \left(\frac{2\pi}{|\mathbf{k}_S|}\right)^{\frac{3-d}{2}} e^{i\frac{3-d}{4}\pi} e^{i\mathbf{k}_S\cdot\mathbf{R}} \sum_{j=1}^{n} \frac{e^{i(\mathbf{k}_I-\mathbf{k}_S)\cdot\mathbf{r}_j}}{|\mathbf{R}-\mathbf{r}_j|^{\frac{d-1}{2}}}. \tag{S3}$$

The far-field measurement allows for the approximation of $|\mathbf{R} - \mathbf{r}_j|^{-(d-1)/2} \sim |\mathbf{R} - \mathbf{r}_c|^{-(d-1)/2}$ where $\mathbf{r}_c = (\sum_{j=1}^{n} \mathbf{r}_j)/n$ is the center of the material. For the wavevector shift $\mathbf{k} = \mathbf{k}_S - \mathbf{k}_I$, the scattering

intensity from n particles then becomes $I_n(\mathbf{k};\,\mathbf{R}) = |\psi_{sc}{}^n(\mathbf{R})|^2 = nI_1(\mathbf{k};\,\mathbf{R})S_n(\mathbf{k})$, where $S_n(\mathbf{k})$ is the static structure factor defined in Eq. (1) in the main text, and $I_1(\mathbf{k};\,\mathbf{R})$ is the scattering intensity from a single particle, which has the following form:

$$I_1(\mathbf{k};\mathbf{R}) = |\psi_I|^2 \, |f(\mathbf{k})|^2 \left(\frac{2\pi}{|\mathbf{k}_S|} \right)^{3-d} \frac{1}{|\mathbf{R}-\mathbf{r}_c|^{d-1}}. \tag{S4}$$

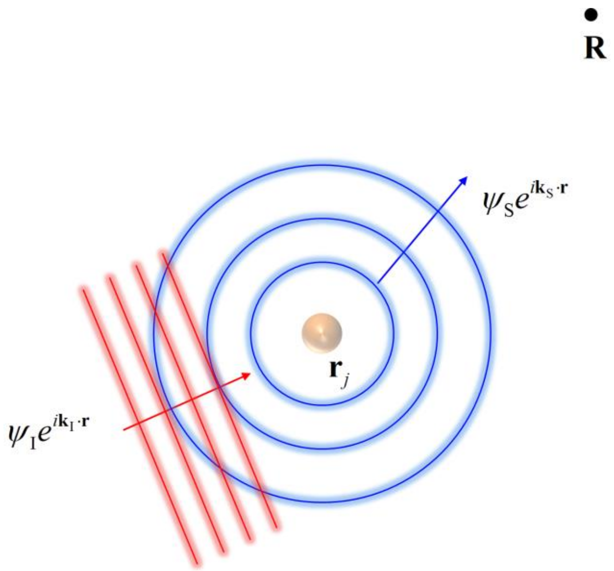

Supplementary Figure 1. Scattering from a particle. Red lines denote the incident planewave and blue lines denote the scattering wave from an excited point source at the jth particle. The scattering field is measured at **R**, which determines the direction of $\mathbf{k}_S$.

Supplementary Note 2. Classification of impulse scattering responses

Consider the impulse scattering response from $S_n(\mathbf{k}) = \delta(\mathbf{k} - \mathbf{k}')$ in two-dimensional (2D) systems, where $\mathbf{k}' = (k_x',k_y')$ (Supplementary Fig. 2). Due to the reciprocity $S_n(\mathbf{k}) = S_n(-\mathbf{k})$, the scattering leads to two $\mathbf{k}'$-shifted light cones of radius $|\mathbf{k}_\mathrm{I}| = k_\mathrm{o}$. The intersections between the original and shifted light cones then determine the allowed scattering wavevectors $\mathbf{k}_\mathrm{S} = (k_x^\mathrm{S},k_y^\mathrm{S})$. The crossing points of these shifted and identical circles are expressed as follows:

$$(k_x^\mathrm{S},k_y^\mathrm{S}) = \frac{1}{2}\left[(k_x',k_y') \pm \sqrt{\left(\frac{2k_\mathrm{o}}{k'}\right)^2 - 1}(k_y',-k_x')\right], \tag{S5}$$

which becomes Eq. (7) in the main text. Notably, because $\mathbf{k}_\mathrm{I} = \mathbf{k}_\mathrm{S}|_{+\mathbf{k}'} - \mathbf{k}' = \mathbf{k}_\mathrm{S}|_{-\mathbf{k}'}$ due to the reciprocity $S_n(\mathbf{k}) = S_n(-\mathbf{k})$, red arrows in Supplementary Fig. 2 and Fig. 1b-d in the main text connected through $\pm\mathbf{k}'$ represent the allowed pairs of the incident and scattering wavevectors.

The angle between the incident and scattering waves is obtained from their inner product $\mathbf{k}_\mathrm{I}\cdot\mathbf{k}_\mathrm{S}$. Because $\mathbf{k}_\mathrm{I} = \mathbf{k}_\mathrm{S} - \mathbf{k}'$ and $\mathbf{k}'\cdot(\mathbf{R}_{\pm\pi/2}\mathbf{k}') = 0$,

$$\begin{aligned}\mathbf{k}_\mathrm{I}\cdot\mathbf{k}_\mathrm{S} &= \frac{1}{4}\left(-\mathbf{k}' + \left[\left(\frac{2k_\mathrm{o}}{k'}\right)^2 - 1\right]^{1/2}\mathbf{R}_{\pm\pi/2}\mathbf{k}'\right)\left(\mathbf{k}' + \left[\left(\frac{2k_\mathrm{o}}{k'}\right)^2 - 1\right]^{1/2}\mathbf{R}_{\pm\pi/2}\mathbf{k}'\right) \\ &= -\frac{1}{4}(k')^2 + \frac{1}{4}\left[\left(\frac{2k_\mathrm{o}}{k'}\right)^2 - 1\right](\mathbf{R}_{\pm\pi/2}\mathbf{k}')\cdot(\mathbf{R}_{\pm\pi/2}\mathbf{k}') \\ &= k_\mathrm{o}^2 - \frac{1}{2}(k')^2.\end{aligned} \tag{S6}$$

The angle θ between $\mathbf{k}_\mathrm{I}$ and $\mathbf{k}_\mathrm{S}$ then satisfies

$$\cos\theta = \frac{\mathbf{k}_\mathrm{I}\cdot\mathbf{k}_\mathrm{S}}{k_\mathrm{o}^2} = 1 - \frac{1}{2}\left(\frac{k'}{k_\mathrm{o}}\right)^2. \tag{S7}$$

When $|\mathbf{k}'| < \sqrt{2}k_\mathrm{o}$, the forward scattering ($0 \le \theta \le \pi/2$ or $3\pi/2 \le \theta \le 2\pi$) is achieved. In contrast,

when $\sqrt{2}k_o \leq |\mathbf{k}'| < 2k_o$, Eq. (S7) gives $\pi/2 \leq \theta \leq 3\pi/2$, which corresponds to the backward scattering. The region of $|\mathbf{k}'| \geq 2k_o$ leads to zero scattering, neglecting too rapid variation of the material landscape under the first-order Born approximation and far-field measurement.

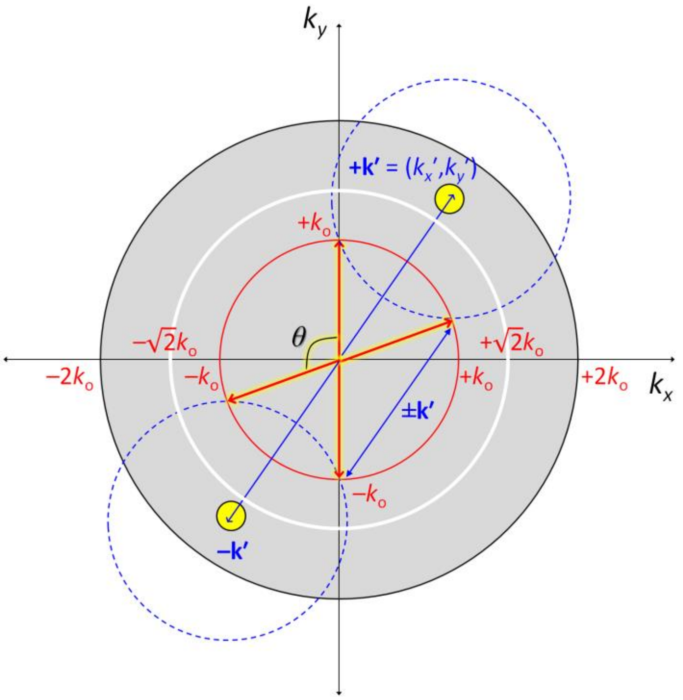

Supplementary Figure 2. Impulse scattering responses. Yellow points denote the impulse on $S(\mathbf{k})$. Red arrows represent the allowed incident and scattering wavevectors from the impulse scattering response $\pm\mathbf{k}'$. Red, white, and black circles have the radii of k_o, $\sqrt{2}k_o$, and $2k_o$, respectively. Blue dashed circles are the shifted light cones due to scattering.

Supplementary Note 3. Evolution model of scattering networks

As shown in previous works[4,5], $S_n(\mathbf{k})$ in Eq. (1) in the main text can be expressed with real-valued parameters, as

$$S_n(\mathbf{k}) = 1 + \frac{2}{n}\sum_{j=1}^{n-1}\sum_{l=j+1}^{n}\cos\left[\mathbf{k}\cdot\left(\mathbf{r}_j - \mathbf{r}_l\right)\right]. \tag{S8}$$

Equation (S8) can be applied to calculate the structure factor $S_{n+1}(\mathbf{k})$ for (n+1)-particle systems, as follows:

$$\begin{aligned} S_{n+1}(\mathbf{k}) &= 1 + \frac{2}{n+1}\sum_{j=1}^{n}\sum_{l=j+1}^{n+1}\cos\left[\mathbf{k}\cdot\left(\mathbf{r}_j - \mathbf{r}_l\right)\right] \\ &= 1 + \frac{2}{n+1}\left(\sum_{j=1}^{n-1}\sum_{l=j+1}^{n}\cos\left[\mathbf{k}\cdot\left(\mathbf{r}_j - \mathbf{r}_l\right)\right] + \sum_{j=1}^{n}\cos\left[\mathbf{k}\cdot\left(\mathbf{r}_j - \mathbf{r}_{n+1}\right)\right]\right) \\ &= 1 + \frac{2}{n+1}\left(\frac{n}{2}\left(S_n(\mathbf{k}) - 1\right) + \sum_{j=1}^{n}\cos\left[\mathbf{k}\cdot\left(\mathbf{r}_j - \mathbf{r}_{n+1}\right)\right]\right) \\ &= \frac{nS_n(\mathbf{k}) + 1}{n+1} + \frac{2}{n+1}\sum_{j=1}^{n}\cos\left[\mathbf{k}\cdot\left(\mathbf{r}_j - \mathbf{r}_{n+1}\right)\right], \end{aligned} \tag{S9}$$

which gives Eqs (8) and (9) in the main text.

The scattering intensity for (n+1)-particle systems is $I_{n+1}(\mathbf{k};\ \mathbf{R}) = (n+1)I_1(\mathbf{k};\ \mathbf{R})S_{n+1}(\mathbf{k})$. Using Eqs (8) and (9) in the main text, the change of the scattering intensity becomes

$$\begin{aligned} I_{n+1}(\mathbf{k}) - I_n(\mathbf{k}) &= (n+1)I_1(\mathbf{k})S_{n+1}(\mathbf{k}) - nI_1(\mathbf{k})S_n(\mathbf{k}) \\ &= I_1(\mathbf{k})\left[(n+1)S_{n+1}(\mathbf{k}) - nS_n(\mathbf{k})\right] \\ &= I_1(\mathbf{k})\left(1 + 2\sum_{j=1}^{n}\cos\left[\mathbf{k}\cdot\left(\mathbf{r}_j - \mathbf{r}_{n+1}\right)\right]\right) \\ &= I_1(\mathbf{k})\left[1 + n\xi_n(\mathbf{k}, \mathbf{r}_{n+1})\right]. \end{aligned} \tag{S10}$$

Supplementary Note 4. Generalization to inhomogeneous materials

Although the evolving scattering network modelling in the main text employs the identical point particle assumption in quantifying the relationship between the structure factor and scattering, the defined network parameters and their evolution process can be directly generalized to scattering from inhomogeneous materials. In this note, we introduce the generalized definitions of the link weight and node degree of evolving scattering networks, the cost function for the evolution process, and their relations to the structure factor and inhomogeneous wave scattering. Suppose a wave equation with an inhomogeneous potential:

$$\nabla^2\psi(\mathbf{r})+\left[V_\mathrm{o}+V_\mathrm{a}(\mathbf{r})\right]\psi(\mathbf{r})=0, \tag{S11}$$

where $\psi(\mathbf{r})$ is the total wave field, and $V_\mathrm{o} = k_\mathrm{o}^2$ and $V_\mathrm{a}(\mathbf{r})$ present the static background potential and spatially varying potential, respectively. Equation (S11) can be applied to wave phenomena governed by the Schrödinger equation in quantum mechanics with inhomogeneous scalar potentials or Maxwell's equations in optics with inhomogeneous permittivity. We restrict the discussion to Hermitian systems with propagating modes by setting the conditions of $V_\mathrm{o} \geq 0$ and $V_\mathrm{o} + V_\mathrm{a}(\mathbf{r}) \geq 0$.

When an incident planewave is set to be $\psi_\mathrm{in}(\mathbf{r}) = \psi_\mathrm{I}\exp(i\mathbf{k}_\mathrm{I}\cdot\mathbf{r})$, the scattering field is defined by $\psi_\mathrm{sc}(\mathbf{r}) = \psi(\mathbf{r}) - \psi_\mathrm{in}(\mathbf{r})$, where its wavevector is $\mathbf{k}_\mathrm{S}$ ($|\mathbf{k}_\mathrm{I}| = |\mathbf{k}_\mathrm{S}| = k_\mathrm{o}$). With the Green's functions in Eq. (S2), the total field at $\mathbf{R}$ satisfies the following Lippmann-Schwinger equation[2]:

$$\psi(\mathbf{R})=\psi_\mathrm{in}(\mathbf{R})+\frac{1}{4\pi}\int G(\mathbf{R},\mathbf{r};\mathbf{k}_\mathrm{S})V_\mathrm{a}(\mathbf{r})\psi(\mathbf{r})d\mathbf{r}=\psi_\mathrm{in}(\mathbf{R})+M\psi(\mathbf{r}), \tag{S12}$$

where the operator M represents the coupling of the total field to the scattering field. When the numerical radius of the operator M is much smaller than $\int|\psi(\mathbf{r})|^2 d\mathbf{r}$, the application of the first-order Born approximation is valid[6]. The recursive form of Eq. (S12) can then be simplified by setting

$M\psi(\mathbf{r}) \sim M\psi_{\text{in}}(\mathbf{r})$, which derives

$$\psi_{\text{sc}}(\mathbf{R}) = \frac{\psi_{\text{I}}}{4\pi}\left(\frac{2\pi}{|\mathbf{k}_{\text{S}}|}\right)^{\frac{3-d}{2}} e^{i\frac{3-d}{4}\pi} e^{i\mathbf{k}_{\text{S}}\cdot\mathbf{R}} \int \frac{V_{\text{a}}(\mathbf{r})e^{i(\mathbf{k}_{\text{I}}-\mathbf{k}_{\text{S}})\cdot\mathbf{r}}}{|\mathbf{R}-\mathbf{r}|^{\frac{d-1}{2}}} d\mathbf{r}. \tag{S13}$$

Same as the derivation in Supplementary Note 1, we assume the far-field measurement with the approximation of $|\mathbf{R} - \mathbf{r}|^{-(d-1)/2} \sim |\mathbf{R} - \mathbf{r}_{\text{c}}|^{-(d-1)/2}$, where $\mathbf{r}_{\text{c}}$ is the center of the entire inhomogeneous material. By defining the wavevector shift $\mathbf{k} = \mathbf{k}_{\text{S}} - \mathbf{k}_{\text{I}}$, the scattering intensity $I_{\text{sc}}(\mathbf{k}; \mathbf{R})$ becomes

$$I_{\text{sc}}(\mathbf{k};\mathbf{R}) = |\psi_{\text{I}}|^2 \frac{1}{16\pi^2}\left(\frac{2\pi}{|\mathbf{k}_{\text{S}}|}\right)^{3-d} \frac{1}{|\mathbf{R}-\mathbf{r}_{\text{c}}|^{d-1}} \left|\int V_{\text{a}}(\mathbf{r})e^{-i\mathbf{k}\cdot\mathbf{r}} d\mathbf{r}\right|^2. \tag{S14}$$

When comparing Eq. (S14) with the identical point particle result of $I_n(\mathbf{k}; \mathbf{R}) = nI_1(\mathbf{k}; \mathbf{R})S_n(\mathbf{k})$ in Supplementary Note 1 and assuming that the entire inhomogeneous potential is confined in the finite-size spatial domain $\mathbf{\Omega}$ (*i.e.*, $V_{\text{a}}(\mathbf{r}) = 0$ if $\mathbf{r} \in \mathbf{\Omega}^{\text{c}}$), we obtain the relationship between scattering and the structure factor defined for an inhomogeneous material:

$$I_{\text{sc}}(\mathbf{k};\mathbf{R}) = |\psi_{\text{I}}|^2 \frac{V_{\Omega}}{16\pi^2}\left(\frac{2\pi}{|\mathbf{k}_{\text{S}}|}\right)^{3-d} \frac{1}{|\mathbf{R}-\mathbf{r}_{\text{c}}|^{d-1}} S(\mathbf{k}), \tag{S15}$$

where V_{Ω} is the volume of the domain $\mathbf{\Omega}$, and the generalized structure factor is

$$S(\mathbf{k}) = \frac{1}{V_{\Omega}}\left|\int_{\Omega} V_{\text{a}}(\mathbf{r})e^{-i\mathbf{k}\cdot\mathbf{r}} d\mathbf{r}\right|^2. \tag{S16}$$

Notably, Eq. (S16) can be simplified to Eq. (1) in the main text when $V_{\text{a}}(\mathbf{r})$ is constant, and the integration is replaced with discretized points. By introducing the cross-correlation function

$$\rho(\mathbf{r}; f, g) = \int_{\Omega} f(\mathbf{r}')g(\mathbf{r}'-\mathbf{r})d\mathbf{r}', \tag{S17}$$

and applying the Wiener–Khinchin theorem to Eq. (S16), $S(\mathbf{k})$ becomes

$$S(\mathbf{k}) = \frac{1}{V_\Omega}\int_\Omega \rho(\mathbf{r};V_\mathrm{a},V_\mathrm{a})e^{-i\mathbf{k}\cdot\mathbf{r}}d\mathbf{r}, \tag{S18}$$

which demonstrates that the structure factor is the Fourier transform of the autocorrelation function of the inhomogeneous potential $V_\mathrm{a}(\mathbf{r})$.

To generalize the evolution process to an inhomogeneous material, we introduce a series of potential modulations $\{\Delta V_{\mathrm{a},n}(\mathbf{r})|\ n = 1, 2, 3, \ldots, N\}$, which is added to V_o. The inhomogeneous potential and the structure factor are defined for each step of modulation, as $V_{\mathrm{a},n}(\mathbf{r}) = \Sigma_n \Delta V_{\mathrm{a},n}(\mathbf{r})$ and $S_n(\mathbf{k}) = (1/V_\Omega)|\int_\Omega V_{\mathrm{a},n}(\mathbf{r})\exp(-i\mathbf{k}\cdot\mathbf{r})d\mathbf{r}|^2 = (1/V_\Omega)\int_\Omega \rho(\mathbf{r};V_{\mathrm{a},n},V_{\mathrm{a},n})\exp(-i\mathbf{k}\cdot\mathbf{r})d\mathbf{r}$. As the generalization of Eq. (S9) to inhomogeneous materials, the evolution of $S_n(\mathbf{k})$ is described as:

$$\begin{aligned} S_{n+1}(\mathbf{k}) &= S_n(\mathbf{k}) + \frac{1}{V_\Omega}\int_\Omega \left[\rho(\mathbf{r};V_{\mathrm{a},n+1},V_{\mathrm{a},n+1}) - \rho(\mathbf{r};V_{\mathrm{a},n},V_{\mathrm{a},n})\right]e^{-i\mathbf{k}\cdot\mathbf{r}}d\mathbf{r} \\ &= S_n(\mathbf{k}) + \frac{1}{V_\Omega}\int_\Omega \left[\rho(\mathbf{r};V_{\mathrm{a},n}+\Delta V_{\mathrm{a},n+1},V_{\mathrm{a},n}+\Delta V_{\mathrm{a},n+1}) - \rho(\mathbf{r};V_{\mathrm{a},n},V_{\mathrm{a},n})\right]e^{-i\mathbf{k}\cdot\mathbf{r}}d\mathbf{r} \\ &= S_n(\mathbf{k}) + \frac{2}{V_\Omega}\mathrm{Re}\left[\int_\Omega \rho(\mathbf{r};\Delta V_{\mathrm{a},n+1},V_{\mathrm{a},n})e^{-i\mathbf{k}\cdot\mathbf{r}}d\mathbf{r}\right] + \frac{1}{V_\Omega}\int_\Omega \rho(\mathbf{r};\Delta V_{\mathrm{a},n+1},\Delta V_{\mathrm{a},n+1})e^{-i\mathbf{k}\cdot\mathbf{r}}d\mathbf{r}, \end{aligned} \tag{S19}$$

or, more concisely, $S_{n+1}(\mathbf{k}) = S_n(\mathbf{k}) + \xi_n(\mathbf{k};\Delta V_{\mathrm{a},n}(\mathbf{r}))$, where

$$\xi_n(\mathbf{k};\Delta V_{\mathrm{a},n+1}(\mathbf{r})) = \frac{2}{V_\Omega}\mathrm{Re}\left[\int_\Omega \rho(\mathbf{r};\Delta V_{\mathrm{a},n+1},V_{\mathrm{a},n})e^{-i\mathbf{k}\cdot\mathbf{r}}d\mathbf{r}\right] + \frac{1}{V_\Omega}\int_\Omega \rho(\mathbf{r};\Delta V_{\mathrm{a},n+1},\Delta V_{\mathrm{a},n+1})e^{-i\mathbf{k}\cdot\mathbf{r}}d\mathbf{r}. \tag{S20}$$

Equation (S20) is the inhomogeneous generalization of Eq. (9) in the main text. When we introduce the identical point particle assumption, the cross-correlation in the first term of the right side of Eq. (S20) becomes the sum of the Dirac delta functions determined by existing particles, which leads to the cosine functions in Eq. (9) in the main text. The second term of the right side of Eq. (S20) is cancelled out for the point particle except for $\mathbf{r} = \mathbf{O}$.

When we revisit the relationship between Eqs (9) and (2) in the main text, the generalized form of the link weight between the pth and q^{th} potential modulations should be

$$w_{p,q}{}^{\mathbf{K}} = \frac{2}{V_{\mathbf{K}}V_{\Omega}}\int_{\mathbf{K}} \mathrm{Re}\left[\int_{\Omega} \rho(\mathbf{r};\Delta V_{\mathrm{a},p},\Delta V_{\mathrm{a},q}) e^{-i\mathbf{k}\cdot\mathbf{r}} d\mathbf{r}\right] d\mathbf{k} \qquad (p \neq q)$$
$$= \frac{1}{V_{\mathbf{K}}V_{\Omega}}\int_{\mathbf{K}}\int_{\Omega} \rho(\mathbf{r};\Delta V_{\mathrm{a},p},\Delta V_{\mathrm{a},q}) e^{-i\mathbf{k}\cdot\mathbf{r}} d\mathbf{r}\, d\mathbf{k} \qquad (p = q). \qquad \text{(S21)}$$

where **K** is the region of interest in the reciprocal space and $V_{\mathbf{K}}$ is the volume of the space **K**. Same as the definition in Eq. (2) in the main text, the link weight in Eq. (S21) is the result of the wave interference induced by different potential modulations if $p \neq q$, though the interference in an inhomogeneous material is determined by the cross-correlation of the potential modulations. There is also a self-loop when $p = q$ case in Eq. (S21), which is expressed as the autocorrelation function due to the finite size of the potentials and becomes constant in the point particle assumption.

Because the self-loop w_{pp} is no longer constant, we define the node degree by including $p = q$, as $w_p{}^{\mathbf{K}} = \sum_q(w_{p,q}{}^{\mathbf{K}})$ for inhomogeneous materials. With a newly defined node degree, Eq. (3) in the main text is generalized to be:

$$\langle S_n \rangle_{\mathbf{K}} = \frac{1}{V_{\mathbf{K}}}\int_{\mathbf{K}} S_n(\mathbf{k}) d\mathbf{k} = \sum_{p=1}^{n} w_p{}^{\mathbf{K}}, \qquad \text{(S22)}$$

which shows that the scattering averaged in the **K** space $\langle S_n \rangle_{\mathbf{K}}$ is directly determined by the sum of the entire node degrees.

When considering the generalized definition of the link weight, node degree, and structure factor listed above, the generalized cost function $\rho_n{}^{\mathbf{K}}(\mathbf{r})$ for the evolution process is

$$\rho_n{}^{\mathbf{K}}(\Delta V_{\mathrm{a}}(\mathbf{r})) = \frac{1}{V_{\mathbf{K}}V_{\Omega}}\sum_{p=1}^{n}\Pi(w_p{}^{\mathbf{K}})$$
$$\times\int_{\mathbf{K}}\left\{2\,\mathrm{Re}\left[\int_{\Omega}\rho(\mathbf{r};\Delta V_{\mathrm{a},p},\Delta V_{\mathrm{a}}(\mathbf{r}))e^{-i\mathbf{k}\cdot\mathbf{r}}d\mathbf{r}\right]+\int_{\Omega}\rho(\mathbf{r};\Delta V_{\mathrm{a}}(\mathbf{r}),\Delta V_{\mathrm{a}}(\mathbf{r}))e^{-i\mathbf{k}\cdot\mathbf{r}}d\mathbf{r}\right\} d\mathbf{k}. \qquad \text{(S23)}$$

At the reciprocal space, Eq. (S23) can be more simplified to be

$$\rho_n^{\mathbf{K}}(\Delta V_\mathrm{a}(\mathbf{r})) = \frac{1}{V_\mathbf{K} V_\Omega} \sum_{p=1}^{n} \Pi(w_p^{\mathbf{K}}) \int_\mathbf{K} \left\{ 2\,\mathrm{Re}\left[\rho(\mathbf{k}; \Delta V_{\mathrm{a},p}, \Delta V_\mathrm{a}(\mathbf{r})) \right] + \rho(\mathbf{k}; \Delta V_\mathrm{a}(\mathbf{r}), \Delta V_\mathrm{a}(\mathbf{r})) \right\} d\mathbf{k}, \quad \text{(S24)}$$

where $\rho(\mathbf{k};f,g)$ is the Fourier transform of the cross-correlation function $\rho(\mathbf{r};f,g)$. The inhomogeneous material design based on the concept of evolving scattering networks is then achieved by engineering the inhomogeneous potential modulation $\Delta V_{\mathrm{a},n}(\mathbf{r})$ to minimize the cost function in Eq. (S24). Notably, the spreading of $\Delta V_{\mathrm{a},n}(\mathbf{r})$ in space and its magnitude can be freely controlled within the limit of the first-order Born approximation.

Table S1 shows the comparison of the scattering and network parameters in the cases of identical point particle assumption and inhomogeneous materials. Despite the slight difference in the coefficients and the notations of node degrees according to the inclusion of the self-loop, the physical meaning of evolving scattering network models is well preserved in inhomogeneous wave scattering. The inhomogeneity characterized by the potential modulation $\Delta V_{\mathrm{a},n}(\mathbf{r})$ imposes the cross- and auto-correlation functions between individual potential modulations on network parameters.

Supplementary Table 1. Generalization of network parameters to inhomogeneous wave scattering. Comparisons of the structure factors $S_n(\mathbf{k})$, link weights $w_{p,q}{}^{\mathbf{K}}$, node degrees $w_p{}^{\mathbf{K}}$, and cost functions $\rho_n{}^{\mathbf{K}}(\mathbf{r})$ or $\rho_n{}^{\mathbf{K}}(\Delta V_{\mathrm{a}}(\mathbf{r}))$ for the evolving scattering network models with identical point particle assumption and generalized inhomogeneous materials.

	Identical point particles	Inhomogeneous materials
$S_n(\mathbf{k})$	Eq. (1)<br>$S_n(\mathbf{k}) = \frac{1}{n}\left\|\sum_{j=1}^{n} e^{-i\mathbf{k}\cdot\mathbf{r}_j}\right\|^2,$	Eq. (S18)<br>$S_n(\mathbf{k}) = \frac{1}{V_\Omega}\int_\Omega \rho(\mathbf{r};V_{\mathrm{a},n},V_{\mathrm{a},n})e^{-i\mathbf{k}\cdot\mathbf{r}}d\mathbf{r}$
$w_{p,q}{}^{\mathbf{K}}$	Eq. (2)<br>$w_{p,q}{}^{\mathbf{K}} = \frac{1}{V_{\mathbf{K}}}\int_{\mathbf{K}}\cos\left[\mathbf{k}\cdot\left(\mathbf{r}_p - \mathbf{r}_q\right)\right]d\mathbf{k}$	Eq. (S21)<br>$p \neq q$:<br>$w_{p,q}{}^{\mathbf{K}} = \frac{2}{V_{\mathbf{K}}V_\Omega}\int_{\mathbf{K}} d\mathbf{k} \times \mathrm{Re}\left[\int_\Omega \rho(\mathbf{r};\Delta V_{\mathrm{a},p},\Delta V_{\mathrm{a},q})e^{-i\mathbf{k}\cdot\mathbf{r}}d\mathbf{r}\right]$<br>$p = q$:<br>$w_{p,q}{}^{\mathbf{K}} = \frac{1}{V_{\mathbf{K}}V_\Omega}\int_{\mathbf{K}} d\mathbf{k} \times \int_\Omega \rho(\mathbf{r};\Delta V_{\mathrm{a},p},\Delta V_{\mathrm{a},q})e^{-i\mathbf{k}\cdot\mathbf{r}}d\mathbf{r}$
$w_p{}^{\mathbf{K}}$	Eq. (3)<br>$\left\langle S_n \right\rangle_{\mathbf{K}} = 1 + \frac{1}{n}\sum_{p=1}^{n} w_p{}^{\mathbf{K}},$	Eq. (S22)<br>$\left\langle S_n \right\rangle_{\mathbf{K}} = \sum_{p=1}^{n} w_p{}^{\mathbf{K}},$
$\rho_n{}^{\mathbf{K}}(\mathbf{r})$	Eq. (4)<br>$\rho_n{}^{\mathbf{K}}(\mathbf{r}) = \frac{1}{nV_{\mathbf{K}}}\sum_{p=1}^{n}\Pi(w_p{}^{\mathbf{K}}) \times \int_{\mathbf{K}}\cos\left[\mathbf{k}\cdot\left(\mathbf{r}_p - \mathbf{r}\right)\right]d\mathbf{k}$	Eq. (S24)<br>$\rho_n{}^{\mathbf{K}}(\Delta V_{\mathrm{a}}(\mathbf{r})) = \frac{1}{V_{\mathbf{K}}V_\Omega}\sum_{p=1}^{n}\Pi(w_p{}^{\mathbf{K}})\int_{\mathbf{K}} d\mathbf{k} \times\left\{2\,\mathrm{Re}\left[\rho(\mathbf{k};\Delta V_{\mathrm{a},p},\Delta V_{\mathrm{a}}(\mathbf{r}))\right] + \rho(\mathbf{k};\Delta V_{\mathrm{a}}(\mathbf{r}),\Delta V_{\mathrm{a}}(\mathbf{r}))\right\},$

Supplementary Note 5. Monte Carlo methods for real and reciprocal spaces

We employ the Monte Carlo method to determine the candidate positions for newly included particles in the 2D finite real space **Ω** around the origin (Supplementary Fig. 3a). The same method is also applied to the reciprocal space **K** to calculate the cost function $\rho_n^{\mathbf{K}}(\mathbf{r})$, though only the region of the first and second quadrants is considered due to the reciprocity $S_n(\mathbf{k}) = S_n(-\mathbf{k})$ (Supplementary Fig. 3b). For statistically homogeneous and isotropic distributions of the sampling points, we set the radial distribution determined by the probability density function proportional to the distance from the origin (|**r**| in **Ω** and |**k**| in **K**, Supplementary Fig. 3c,d). The spacing along the angular axis is set to be regular in both cases. An ensemble of realizations studied in the main text is then obtained with different realizations of Monte Carlo samplings having the same stochastic distributions in **Ω** and **K**. The method can also be applied to 3D systems by using the probability density function proportional to the square of the distance.

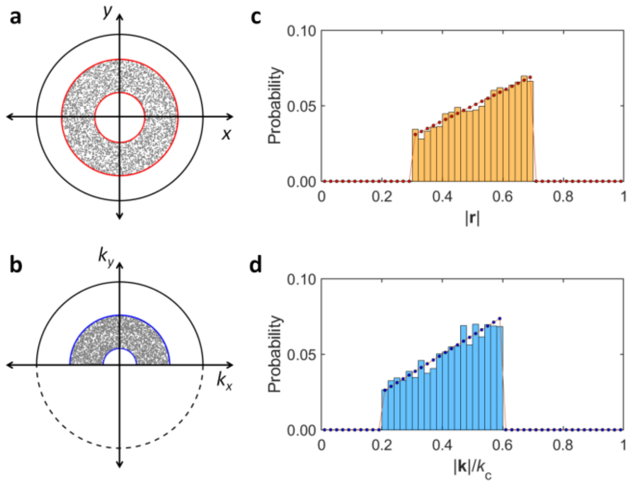

Supplementary Figure 3. Monte Carlo methods for discretization. a,b, An example of discretization for ring-shaped real and reciprocal spaces: **a,** candidate positions in the real space **Ω**, and **b,** reciprocal-space states for estimating the cost function. **c,d,** Probability distributions for **c,** real and **d,** reciprocal spaces, showing the density proportional to the distance from the origin. **a-d** describes an example of the employed Monte Carlo method with the ring-shaped **Ω** and **K**.

Supplementary Note 6. Evolution process using position as a resource

Supplementary Figure 4 shows the evolution of the cost function $\rho_n^{\mathbf{K}}(\mathbf{r})$, showing the effect of wave interferences and the finite real space $\mathbf{\Omega}$. While a new particle is located at the position minimizing $\rho_n^{\mathbf{K}}(\mathbf{r})$ (yellow region), multiple interferences from the reciprocal space $\mathbf{K}$ decrease the region of large $|\rho_n^{\mathbf{K}}(\mathbf{r})|$ (evolution from Supplementary Fig. 4a to 4h). The result demonstrates that the evolution process consumes the real space as a resource for suppressing wave scattering.

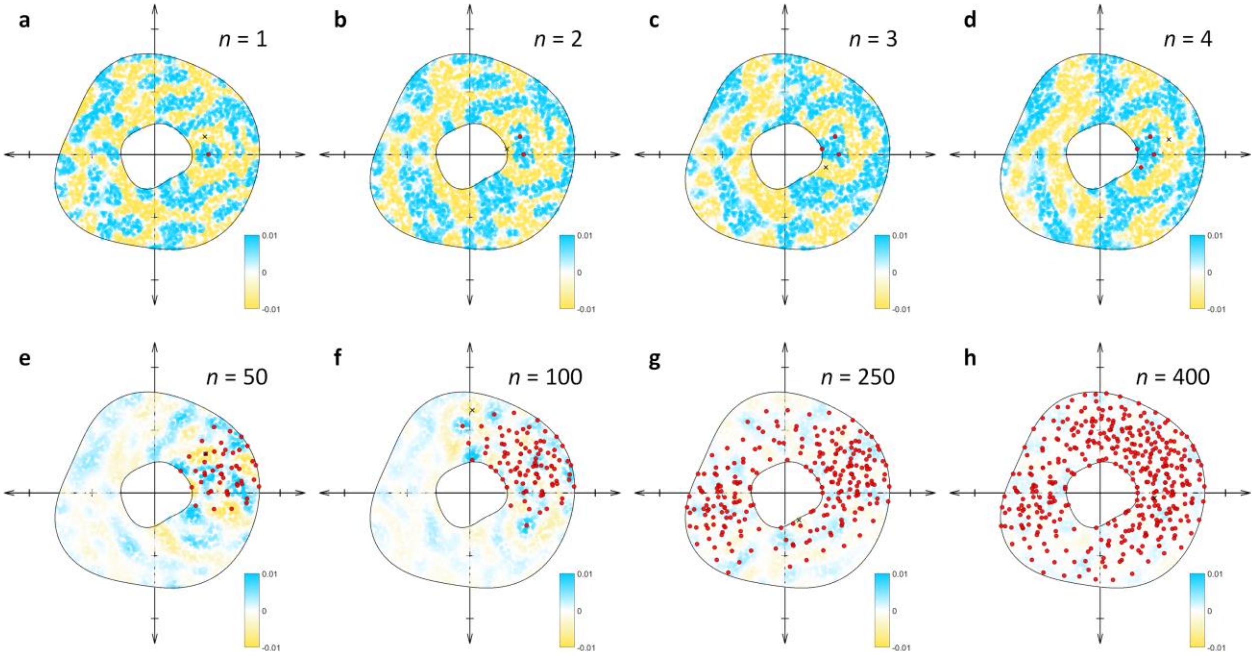

Supplementary Figure 4. Evolution of the cost function. a-h, The evolution of $\rho_n^{\mathbf{K}}(\mathbf{r})$ at **a,** n = 1, **b,** n = 2, **c,** n = 3, **d,** n = 4, **e,** n = 50, **f,** n = 100, **g,** n = 250, and **h,** n = 400. Red markers are particles. Yellow and blue regions denote the spaces of negative and position $\rho_n^{\mathbf{K}}(\mathbf{r})$, respectively. The '×' marker represents the position $\mathbf{r}_{\min}$ of $\rho_n^{\mathbf{K}}(\mathbf{r}_{\min}) = \min[\rho_n^{\mathbf{K}}(\mathbf{r})]$, showing the location of the next particle. Supplementary Movie S1 describes the entire evolution of the n = 500 scattering network. The result is based on the evolution process in Fig. 2 in the main text.

Supplementary Note 7. Performance analysis of the evolution process

The performance of the evolution process is characterized by the evolution of $\rho_n^{\mathbf{K}} = \min[\rho_n^{\mathbf{K}}(\mathbf{r})]$ and the average structure factor $\langle S_n \rangle_{\mathbf{K}}$ over the reciprocal space **K**. In line with the result in Supplementary Fig. 4, Supplementary Fig. 5a also shows that the consumed finite real space by including particles leads to the decrease of the amount of suppression in scattering. Supplementary Figure 5b demonstrates that the Monte Carlo discretization successfully develops the target evolution of a scattering network, as shown in the similar suppression in the Monte Carlo result (blue markers) and the validation result (red markers).

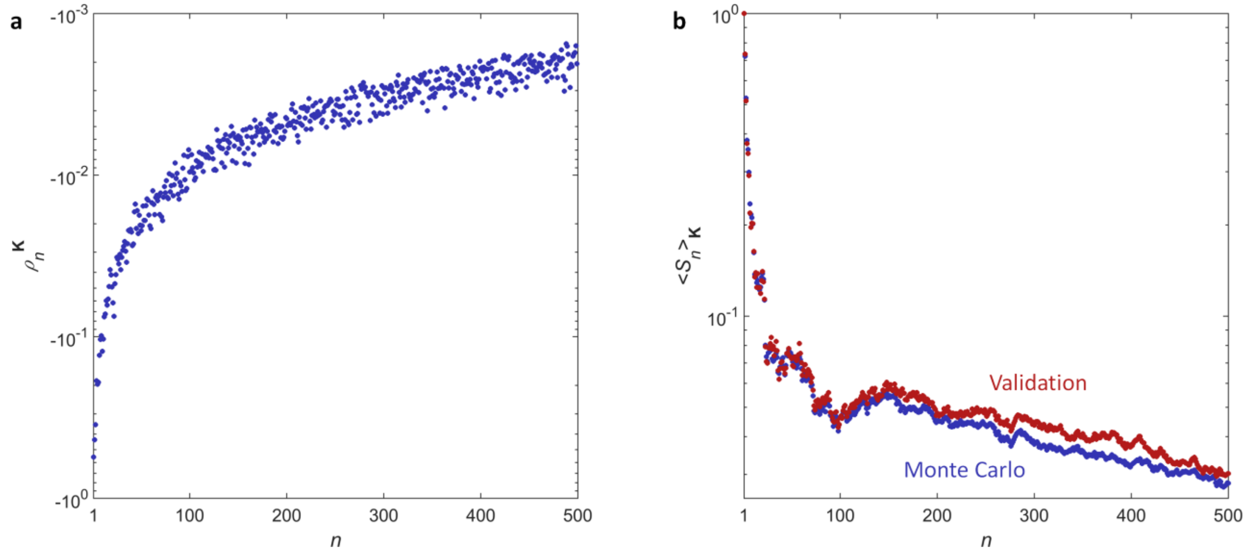

Supplementary Figure 5. Performance of the evolution process. a, The evolution of the minimum cost function $\min[\rho_n^{\mathbf{K}}(\mathbf{r})]$. **b,** The evolution of the average structure factor $\langle S_n \rangle_{\mathbf{K}}$. Blue markers in **b** represent $\langle S_n \rangle_{\mathbf{K}}$ for the reciprocal states from the Monte Carlo discretization of 1200 points, which corresponds to the optimization result for the training data. Red markers in **b** represent $\langle S_n \rangle_{\mathbf{K}}$ for the reciprocal states from the regular discretization of 7948 points, which corresponds to the optimization result for the validation data. The result is based on the evolution process in Fig. 2 in the main text.

Supplementary Note 8. Perturbed structure factor from random defects

Suppose that the evolution process leads to a set of the particle positions $R_o = \{\mathbf{r}_1, \mathbf{r}_2, \ldots, \boldsymbol{r}_n\}$ that provides the structure factor $S_n^{o}(\mathbf{k})$. From the set R_o, we also introduce another set of the particle positions with defects $R_d = \{\mathbf{r}_1 + \Delta\mathbf{r}_1, \mathbf{r}_2 + \Delta\mathbf{r}_2, \ldots, \boldsymbol{r}_n + \Delta\mathbf{r}_n\}$. From Eq. (S8), the structure factor $S_n^{d}(\mathbf{k})$ for the defected set R_d is

$$S_n^{\,\mathrm{d}}(\mathbf{k}) = S_n^{\,\mathrm{o}}(\mathbf{k}) + \frac{2}{n}\sum_{j=1}^{n-1}\sum_{l=j+1}^{n}\left(\cos\left[\mathbf{k}\cdot\left(\mathbf{r}_j + \Delta\mathbf{r}_j - \mathbf{r}_l - \Delta\mathbf{r}_l\right)\right] - \cos\left[\mathbf{k}\cdot\left(\mathbf{r}_j - \mathbf{r}_l\right)\right]\right). \qquad \text{(S25)}$$

The perturbation of the structure factor then becomes

$$\left|S_n^{\,\mathrm{d}}(\mathbf{k}) - S_n^{\,\mathrm{o}}(\mathbf{k})\right| = \frac{4}{n}\left|\sum_{j=1}^{n-1}\sum_{l=j+1}^{n}\sin\left[\mathbf{k}\cdot\left(\mathbf{r}_j - \mathbf{r}_l + \frac{\Delta\mathbf{r}_j - \Delta\mathbf{r}_l}{2}\right)\right]\sin\left[\mathbf{k}\cdot\left(\frac{\Delta\mathbf{r}_j - \Delta\mathbf{r}_l}{2}\right)\right]\right|. \qquad \text{(S26)}$$

When the defects are sufficiently small enough, Eq. (S26) can be approximated to be:

$$\left|S_n^{\,\mathrm{d}}(\mathbf{k}) - S_n^{\,\mathrm{o}}(\mathbf{k})\right| \sim \frac{4}{n}\left|\sum_{j=1}^{n-1}\sum_{l=j+1}^{n}\sin\left[\mathbf{k}\cdot\left(\mathbf{r}_j - \mathbf{r}_l\right)\right]\left[\mathbf{k}\cdot\left(\frac{\Delta\mathbf{r}_j - \Delta\mathbf{r}_l}{2}\right)\right]\right|. \qquad \text{(S27)}$$

In the SHU design, the cost function enforces the finding of $\cos[\mathbf{k}\cdot(\mathbf{r}_j - \mathbf{r}_l)] \rightarrow -1$, which gives $\sin[\mathbf{k}\cdot(\mathbf{r}_j - \mathbf{r}_l)] \rightarrow 0$. Therefore, the particles with highly negative node degrees $w_p^{\mathbf{K}}$, which correspond to more critical particles in the SHU implementation, possess stronger defect immunity.

Supplementary Note 9. Non-preferential attachment for suppressed scattering

From Eqs (2) and (4) in the main text, non-preferential attachment of $\Pi(w_p^{\mathbf{K}}) = 1$ leads to a clear connection between the n+1th node degree and the cost function with $\min[\rho_n^{\mathbf{K}}(\mathbf{r})] = \rho_n^{\mathbf{K}}(\mathbf{r}_{\min})$, as follows:

$$\begin{aligned}\rho_n^{\mathbf{K}}(\mathbf{r}_{\min} = \mathbf{r}_{n+1}) &= \frac{1}{n}\sum_{p=1}^{n}\left[\frac{1}{V_{\mathbf{K}}}\int_{\mathbf{K}}\cos\left[\mathbf{k}\cdot\left(\mathbf{r}_p - \mathbf{r}_{n+1}\right)\right]d\mathbf{k}\right] \\ &= \frac{1}{n}\sum_{p=1}^{n} w_{p,n+1}^{\mathbf{K}} = \frac{w_{n+1}^{\mathbf{K}}}{n}.\end{aligned} \tag{S28}$$

With Eq. (3), we can also characterize the impact of the n+1th node degree on the change of the average structure factor from $\langle S_n \rangle_{\mathbf{K}}$ to $\langle S_{n+1} \rangle_{\mathbf{K}}$:

$$w_{n+1}^{\mathbf{K}} = (n+1)\left(\langle S_{n+1}\rangle_{\mathbf{K}} - 1\right) - n\left(\langle S_n\rangle_{\mathbf{K}} - 1\right). \tag{S29}$$

Equations (S28) and (S29) result in Eq. (5) in the main text.

Supplementary Note 10. Degree distributions in the crystals of different *n*

With discrete translational symmetry, square-lattice crystals of the x and y periodicity Λ have the first Bragg peak at $\mathbf{k}_{\mathrm{Bragg}} = \pm(2\pi / \Lambda)\mathbf{e}_x$ and $\pm(2\pi / \Lambda)\mathbf{e}_y$, where $\mathbf{e}_{x,y}$ are x- and y-axis unit vectors. Changing Λ thus alters scattering responses in the reciprocal spaces of interest $\mathbf{K}_{\mathrm{L}}$ and $\mathbf{K}_{\mathrm{S}}$. We employ $n = 149$ with $\Lambda = 1/7$ in the main text to obtain scattering responses similar to those of evolving materials: $S_n(\mathbf{k}\in\mathbf{K}_{\mathrm{L}}) \sim 0$ and $S_n(\mathbf{k}\in\mathbf{K}_{\mathrm{S}}) >> 0$. For comparison, in this section, we investigate the degree distributions of the crystals of different n.

Supplementary Figure 6a-c shows the real-space particle distributions and the reciprocal-space structure factors of the crystals of $n = 253$ ($\Lambda = 1/9$), $n = 377$ ($\Lambda = 1/11$), and $n = 529$ ($\Lambda = 1/13$). More particles in a given space, which result in a smaller characteristic length, lead to a larger magnitude of $\mathbf{k}_{\mathrm{Bragg}}$. Because $S_n(|\mathbf{k}| \leq |\mathbf{k}_{\mathrm{Bragg}}|) \sim 0$, the change of n eventually alters the scattering at the short-range scale when we fix the value of kth for the comparison with evolving materials. As shown in the node degree distributions (Supplementary Fig. 6d-i), the increase of $|\mathbf{k}_{\mathrm{Bragg}}|$ provides the negative node degree at the short-range scale $\mathbf{K}_{\mathrm{S}}$ (Supplementary Fig. 6g-i), while the original SHU condition is preserved (Supplementary Fig. 6d-f). Especially, as shown in the result of $n = 529$, which has the first Bragg peak $|\mathbf{k}_{\mathrm{Bragg}}| = 1.163k_{\mathrm{c}}$, the SHU materials with a similar particle number ($n = 500$) support stronger scattering in $\mathbf{K}_{\mathrm{S}}$. For comparison, the illustration of microstructures using node degrees is also presented in Supplementary Fig. 6j-l, showing that the contribution of each particle is nonuniform in the finite structures, even in crystals.

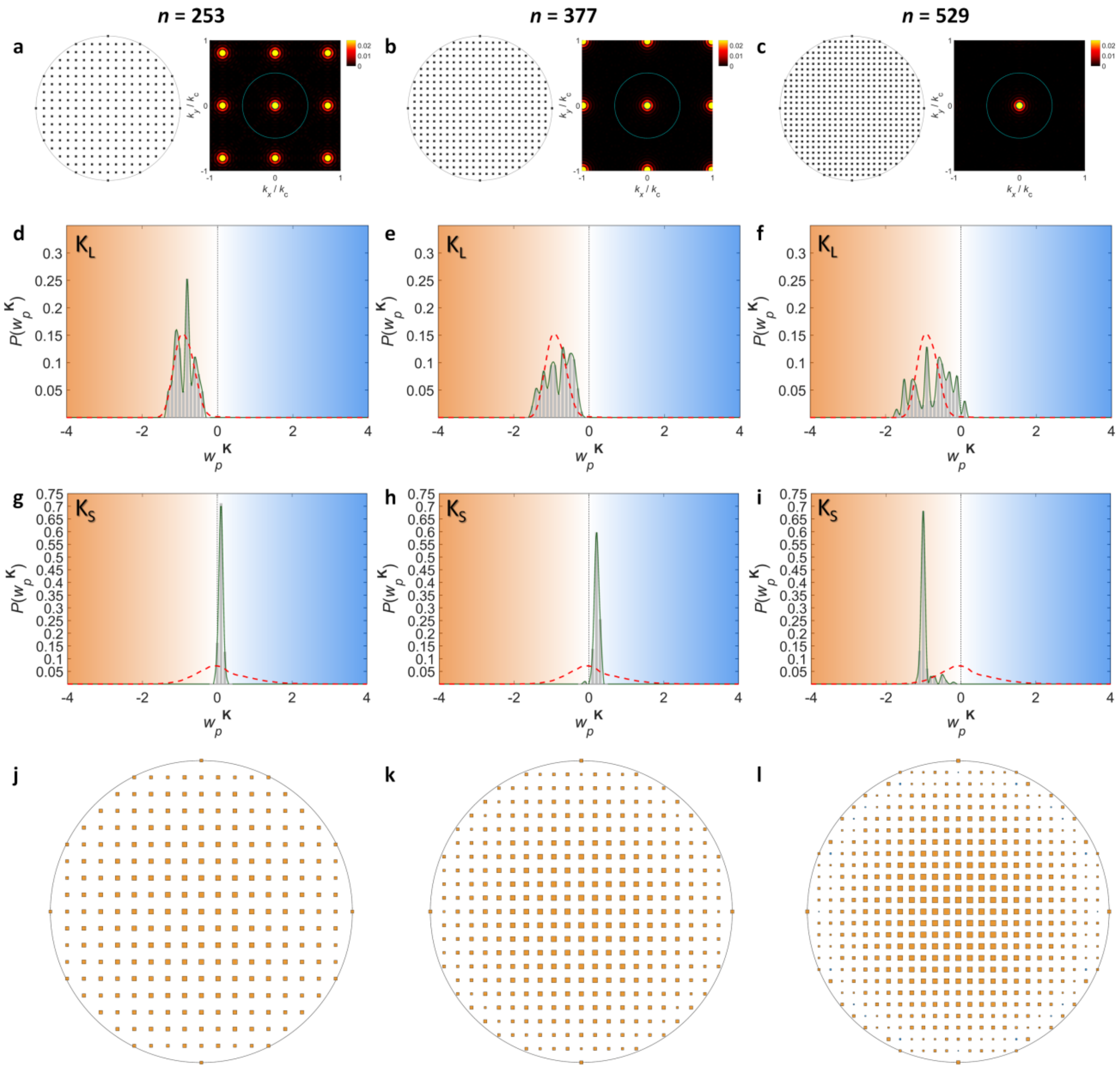

Supplementary Figure 6. Crystals of different particle numbers. Different n crystals are analyzed: n = 253 with Λ = 1/9 (**a,d,g,j**), n = 377 with Λ = 1/11 (**b,e,h,k**), and n = 253 with Λ = 1/9 (**c,f,i,l**). **a-c,** Material phase and the resulting structure factor. **d-i,** Node degree distributions for the **d-f,** long-range scale with $\mathbf{K} = \mathbf{K_L}$ and **g-i**, short-range scale with $\mathbf{K} = \mathbf{K_S}$, where $P(w_p^{\mathbf{K}})$ represents the probability density distribution. The red and blue background colors represent negative (or suppressing) and positive (or enhancing) contributions to scattering, respectively. The red dashed lines are the results of Figs 3e and 3h. **j-l,** Visualizations of materials with the node degrees for $\mathbf{K_L}$, where the orange and blue markers denote the negative and positive node degrees, respectively. The size of a marker represents the magnitude of the node degree $|w_p^{\mathbf{K_L}}|$.

Supplementary Note 11. Evolution of scattering in Poisson and evolving SHU materials

Supplementary Figure 7 represents the evolution of structure factors (or scattering) averaged in the reciprocal spaces of different length scales: $<S_n>_{\mathbf{K_L}}$ for long-range and $<S_n>_{\mathbf{K_S}}$ for short-range scales. In Poisson materials (Supplementary Fig. 7a), the short-range scattering maintains its initial level. In contrast, the long-range scattering increases as the Poisson process gradually fills the finite real space. In evolving SHU materials (Supplementary Fig. 7b), while the short-range scattering again maintains a similar level to the initial state, the long-range scattering is suppressed rapidly. Due to the finite real space, the suppression continues until $n \sim 150$, which is close to the number of particles of the crystal ($n = 149$) having the first Bragg peak $|\mathbf{k}_{\mathrm{Bragg}}| = 0.626k_{\mathrm{c}}$. Although the crystal of $n = 529$ has a very small value of $<S_n>_{\mathbf{K_S}}$ (Supplementary Fig. 6c,f,i), the SHU evolution process maintains the values of both long-range and short-range orders after $n \sim 150$. The result shows the uniqueness of SHU materials, allowing the increase of particle density (from $n \sim 150$ to $n = 500$) while preserving crystal-like scattering ($n = 149$).

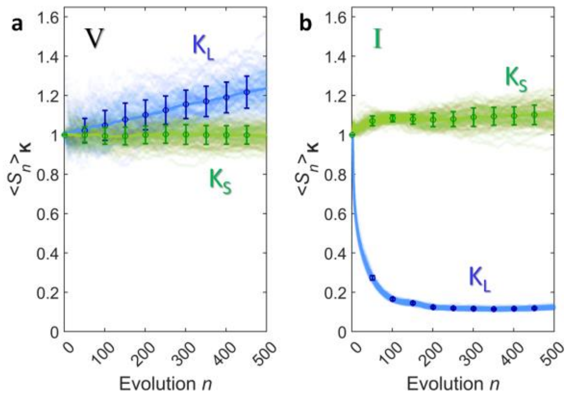

Supplementary Figure 7. Evolutions of structure factors at different length scales. a,b, Evolutions of $<S_n>_{\mathbf{K_L}}$ (blue points) and $<S_n>_{\mathbf{K_S}}$ (green points) during the Poisson and SHU processes of Fig. 3 in the main text. A random ensemble of 100 realizations is investigated for both **a,** Poisson and **b,** SHU processes. Circles and error bars represent the mean value and one standard deviation of each ensemble of 100 realizations. The symbols 'V' and 'I' represent the phases of Poisson and SHU materials, which are discussed in Fig. 6 in the main text.

Supplementary Note 12. Extended data and discussion for evolving material screening

Supplementary Figure 8 shows the evolutions of averaged scatterings in different length scales $<S_n>_{\mathbf{K_L}}$ and $<S_n>_{\mathbf{K_S}}$ for the core and cladding configurations. In all cases, the enhanced SHU screening of Poisson materials by using evolving scattering networks is evident, as demonstrated in the suppressed $<S_n>_{\mathbf{K_L}}$ in Supplementary Fig. 8b,d compared to Supplementary Fig. 8a,c.

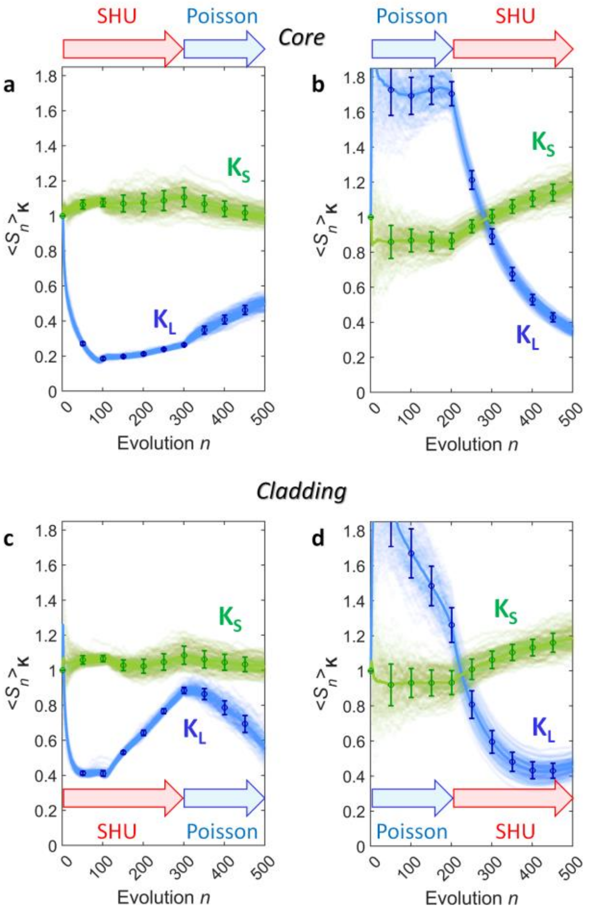

Supplementary Figure 8. Effect of structural configurations on material screening. Evolutions of $<S_n>_{\mathbf{K_L}}$ (blue points) and $<S_n>_{\mathbf{K_S}}$ (green points) during the processes for the **a,b,** core and **c,d,** cladding configurations in Fig. 4b,e and Fig. 4c,f in the main text, respectively. A random ensemble of 100 realizations is investigated for both **a,c,** SHU→Poisson and **b,d,** Poisson→SHU sequences. Circles and error bars represent the mean value and one standard deviation of each ensemble of 100 realizations.

In Supplementary Fig. 8, two essential features in evolving material screening are observed. First, the structural configuration is strongly related to the screening performance, though evolving scattering networks always provide better performance. For example, superior material screening is obtained with the mixing and core configurations compared to the cladding configuration. This result can be explained with the network viewpoint, showing the unequal importance of the positions in the finite real space. As shown in Fig. 3k,l in the main text, the particles near the center provide more contributions to scattering than the outside ones, leading to more efficient SHU screening in the core configuration than in the cladding one. Second, the short-range scattering $<S_n>_{\mathbf{K_S}}$ of evolving scattering networks (Supplementary Fig. 8b,d and Fig. 4h in the main text) has larger values than that of a simple combination of the material states (Supplementary Fig. 8a,c and Fig. 4g in the main text) as the result of suppression of the long-range scattering $<S_n>_{\mathbf{K_L}}$.

In screening material properties using evolving scattering networks, the ratio between Poisson and SHU processes is an essential factor in determining the screening performance. Supplementary Figure 9 shows a case in which material screening is more accessible compared to Supplementary Fig. 4 in the main text: $n = 500$ particles in a single realization, which is composed of 400 particles obtained from the SHU process and 100 particles obtained from the Poisson process. In order to maintain the statistical density of particles over the entire space, we divide the real space into $\mathbf{\Omega}$ (red shaded area) and $\mathbf{\Omega}^c$ (blue shaded area) with the circle boundaries of the radii 0.8944 and 0.4472 for the core and cladding configurations, respectively. As expected, the final results after the screening have more complete forms of SHU states, as shown in reduced blue markers in Supplementary Fig. 9d-f compared to Fig. 4d-f in the main text.

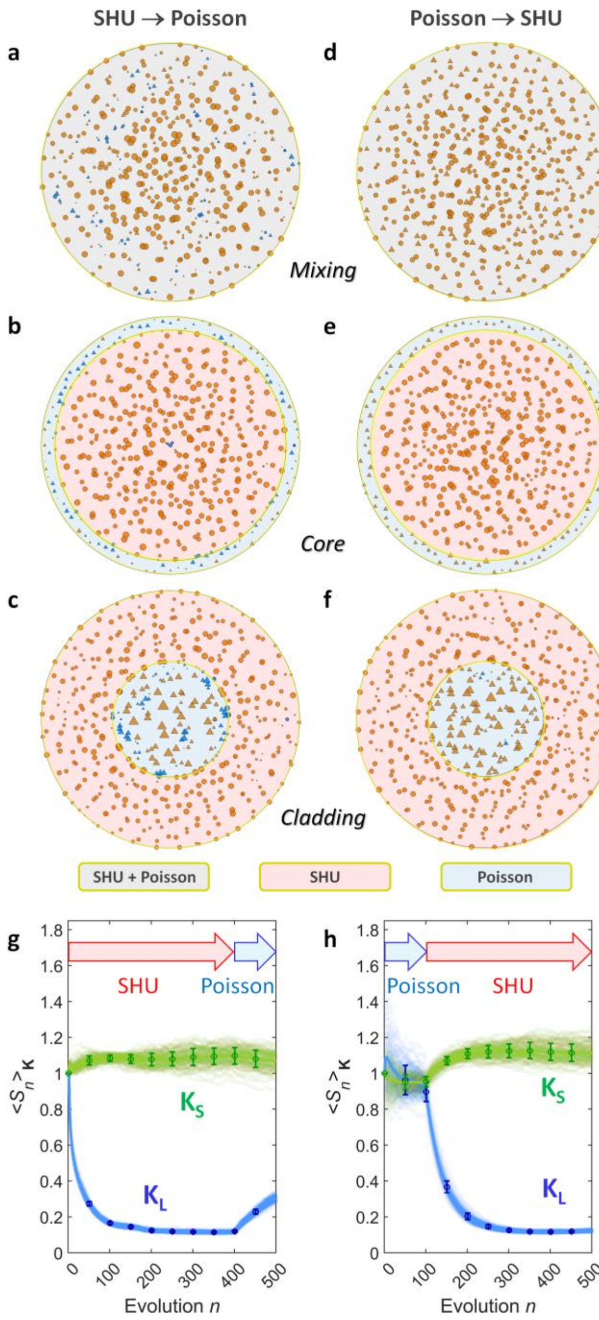

Supplementary Figure 9. Effect of the ratio between Poisson and SHU processes. a-f, Visualizations of materials with the node degrees for $\mathbf{K_L}$, where the orange and blue markers denote the negative and positive node degrees, respectively: **a-c,** SHU→Poisson processes, and **d-f,** Poisson→SHU processes. The size of a marker represents the magnitude of the node degree $|w_p^{\mathbf{K_L}}|$. Among n = 500 particles for each realization, SHU and Poisson processes cover 400 and 100 particles, respectively. The realizations in **a-f** are classified according to the allowed region of

the real space for each process: **a,d,** SHU mixing configuration sharing the entire space for SHU and Poisson processes (grey shaded area), **b,e,** SHU core configuration with an inner SHU region (red shaded area) and outer Poisson region (blue shaded area), and **c,f,** SHU cladding configuration with an inner Poisson region (blue shaded area) and outer SHU region (red shaded area). **g,h,** Evolutions of $<S_n>_{\mathbf{K_L}}$ (blue points) and $<S_n>_{\mathbf{K_S}}$ (green points) during the processes for the mixing configuration in **a,d**. A random ensemble of 100 realizations is investigated for both **g,** SHU$\rightarrow$Poisson and **h,** Poisson$\rightarrow$SHU sequences. In **g** and **h**, circles and error bars represent the mean value and one standard deviation of each ensemble of 100 realizations.

By using the link weights $w_{p,q}^{\mathbf{K}}$, the architecture of a designed evolving network can be visualized. Supplementary Figure 10 shows the network architectures of Fig. 4a-f in the main text. Because each network is fully connected, we present the 1250 most important links (0.5 % of the entire links) for the SHU realization due to their highly negative values. When comparing three different screening configurations—Mixing, Core, and Cladding—the plotted links are concentrated between the particles in the SHU regions, as shown in the comparison of Supplementary Fig. 10a,d, 10b,e, and 10c,f.

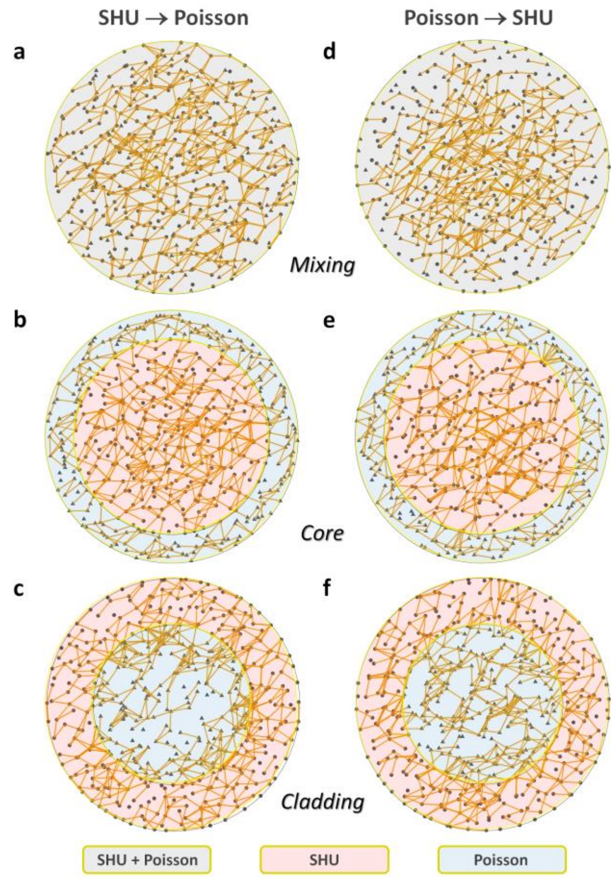

Supplementary Figure 10. Network architectures of material screening. a-f, Network visualizations of materials using the link weights $w_{p,q}^{\mathbf{K_L}}$: **a-c,** SHU→Poisson processes, and **d-f,** Poisson→SHU processes. Among n = 500 particles for each realization, SHU and Poisson processes cover 300 and 200 particles, respectively. The screening configurations are the same as those in Fig. 4a-f in the main text. For each case, 1250 links with the smallest weight values are plotted.

Supplementary Note 13. Evolution and network architecture with preferential attachment

Supplementary Figure 11 shows the evolutions of averaged scatterings in different length scales for the Poisson process and the SHU process with preferential and anti-preferential attachment. While the Poisson process shows a simple evolution with increasing long-range scattering and preserved short-range scattering (Supplementary Fig. 11a), all the SHU processes first show the significant suppression of the long-range scattering (Supplementary Fig. 11b-d) until the first branch point (n_{B1}, black triangle). After n_{B1}, preferential and anti-preferential attachment shows the opposite behaviour in terms of short-range scattering: suppressed $<S_n>_{\mathbf{K_S}}$ with preferential attachment (Supplementary Fig. 11b,c) and enhanced $<S_n>_{\mathbf{K_S}}$ with anti-preferential attachment (Supplementary Fig. 11d) until the second branch point (n_{B2}, red triangle). After n_{B2}, the suppression of long-range and short-range scattering breaks down in strong preferential attachment (Supplementary Fig. 11c), leading to the separation of phases II and III. We note that the second branch point n_{B2} depends on the strength of preference, that is, n_{B2} is a function of α in the example of the tangent hyperbolic preference function $\Pi(w_p^{\mathbf{K}})$.

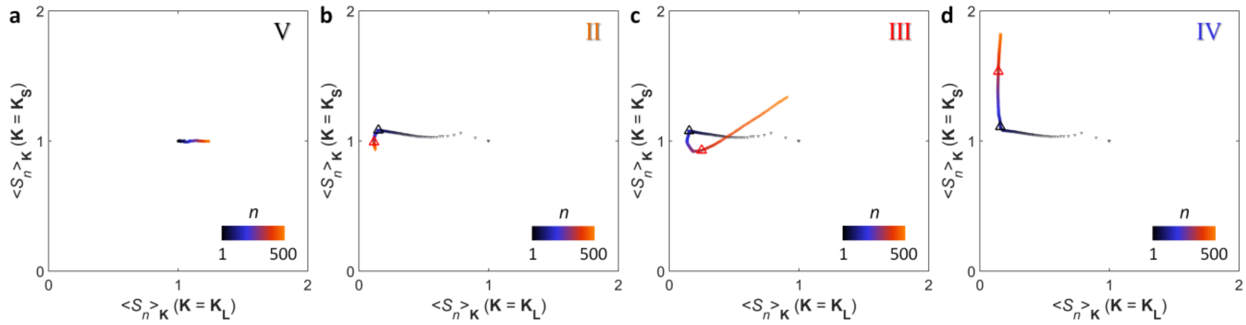

Supplementary Figure 11. Evolution of scattering for different material phases. The evolution of ensemble-averaged long-range and short-range scatterings $<S_n>_{\mathbf{K_L}}$ and $<S_n>_{\mathbf{K_S}}$ with the **a,** Poisson process (phase V), **b,** weak preferential attachment (phase II, α = +0.2), **c,** strong preferential attachment (phase III, α = +0.6), and **d,** strong anti-preferential attachment (phase IV, α = –0.6). Each marker denotes $<S_n>_{\mathbf{K_L}}$ and $<S_n>_{\mathbf{K_S}}$ averaged for a random ensemble of 100

realizations. The color of the marker represents the evolving particle number n. Black and red triangles represent the first and second branch points n_{B1} and n_{B2}.

For reference, examples of the network architectures of different material phases I-IV are shown in Supplementary Fig. 12. Similar to Supplementary Fig. 10, the 1250 most important links (0.5 % of the entire links) for the SHU realization are presented. Supplementary Figure 12d for the material phase IV, which has much larger $<S_n>_{\mathbf{K_S}}$ than the other phases, also shows unique network architectures when compared with Supplementary Fig. 12a-c.

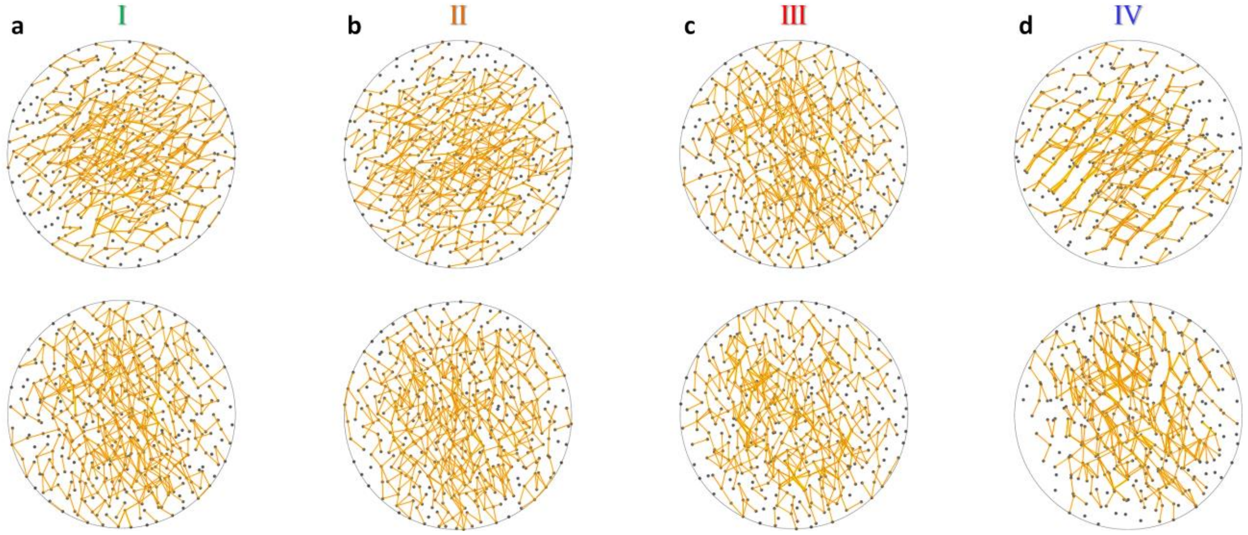

Supplementary Figure 12. Network architectures of different material phases. a-d, Network visualizations of materials using the link weights $w_{p,q}^{\mathbf{K_L}}$: **a,** phase I, **b,** phase II, **c,** phase III, and **d,** phase IV. The top and bottom figures in each case represent two random realizations of materials. For each case, 1250 links with the smallest weight values are plotted.

Supplementary Note 14. Full-wave calculation for evolving scattering networks

To verify the analysis of this work, we calculate the structure factors for the designed evolving scattering networks through the full-wave simulation. The simulation is conducted with the RF module of the COMSOL Multiphysics for 2D in-plane transverse electric modes. For the characteristic wavelength of the simulation λ_{sim} = 1500 nm ($k_{\text{sim}} = 2\pi/\lambda_{\text{sim}} = 4.19\times10^6$ m^{-1}), the finite-range real space $\mathbf{\Omega}$ that contains 500 particles is set to be a circle with a radius of $6\lambda_{\text{sim}}$ = 9 μm and a free-space relative permittivity of 1. The upper limit of the spatial frequency of interest determined by the number of particles inside $\mathbf{\Omega}$ (see Methods) is then $k_c = 7.81\times10^6$ m^{-1}, which corresponds to the characteristic distance of d_c = 805 nm. The identical nonmagnetic particles are assumed to have a radius of $0.20\lambda_{\text{sim}}$ = 300 nm and a relative permittivity of 1.5. This condition satisfies the first-order Born approximation roughly for the free-space optical wavelength of $\lambda_o > 0.56\lambda_{\text{sim}}$ = 847 nm (or, $k_o = 2\pi/\lambda_o < 1.77k_{\text{sim}} = 0.95k_c$).

For the experimental verification, the useful form of the structure factor is:

$$S_n(\mathbf{k}) = \frac{I_n(\mathbf{k};\mathbf{R})}{nI_1(\mathbf{k};\mathbf{R})}, \tag{S30}$$

which is based on comparing the scattering intensities from a many-particle material and a single particle at the constant measuring points (constant $|\mathbf{R} - \mathbf{r}_c|$). To obtain $I_n(\mathbf{k};\mathbf{R})$ and $I_1(\mathbf{k};\mathbf{R})$ in Eq. (S30), we set the circle of the measuring points at $10\lambda_{\text{sim}}$ = 15 μm (Supplementary Fig. 13a) for the planewave incidence condition. The scattering field is measured for the particle distributions designed in the main text (Supplementary Fig. 13b,c). By controlling the angles of the incident waves ($0 \le \theta_I < 2\pi$) and the measuring points ($0 \le \theta_S < 2\pi$), and the optical frequency to manipulate $|\mathbf{k}_I| = |\mathbf{k}_S| = k_o$, all the 2D reciprocal space $\mathbf{k} = \mathbf{k}_S - \mathbf{k}_I$ for the structure factor can be examined. For the comparison with the results in the main text, we conduct the simulation of $0.02k_c \le k_o \le 0.80k_c$

with the interval $0.02k_c$, which allows for the calculation of the structure factor in the region of $|\mathbf{k}| \leq 1.60k_c$.

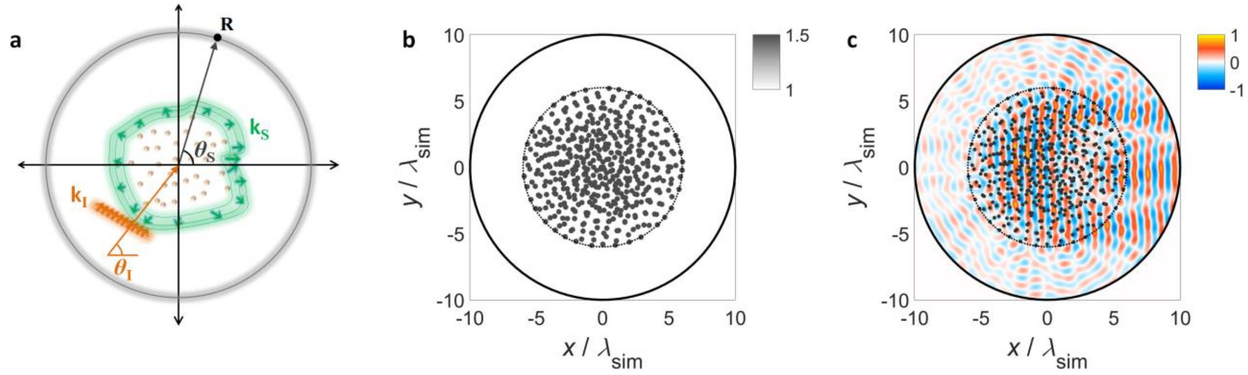

Supplementary Figure 13. Full-wave analysis of scattering from evolving scattering networks. **a,** A schematic of the setup for full-wave analysis. In the simulation, the angles for the incident waves ($0 \leq \theta_I < 2\pi$) and the measuring points ($0 \leq \theta_S < 2\pi$) are discretized with the intervals $2\pi/50$ and $2\pi/200$, respectively. **b,c,** Examples of the profiles of **b,** relative permittivity and **c,** scattering field. Solid and dashed circles denote the radii of $10\lambda_{sim}$ and $6\lambda_{sim}$, respectively. The perfectly matched layer is applied to the boundary condition outside the solid circle.

Supplementary Figure 14 shows the comparison between the results from the full-wave simulation (Supplementary Fig. 14a-c) and point particle assumption (Supplementary Fig. 14d-f) for different phases of material in Fig. 6 in the main text. As shown, the point particle assumption developed in the main text shows good agreement with the results from the full-wave analysis. In detail, the regime of insuppressible scattering near the infinite wavelength increases ($k_{min} \sim 0.15k_c$) due to non-monopole radiation that results in $I_1(\mathbf{k}; \mathbf{R}) \sim 0$ and thus the singularity in Eq. (S30), which leads to the increase near $|\mathbf{k}| \sim 0$ during the interpolation. However, the apparent distinction between the SHU states (Supplementary Fig. 14a,b) and the Poisson state (Supplementary Fig. 14c) is evident despite the finite size of particles. When comparing Supplementary Figs 14a and 14b, the increase of long-range scattering $\langle S_n \rangle_{\mathbf{K_L}}$ in the material phase IV (Supplementary Fig. 14b,e) is successfully demonstrated. For a more rigorous analysis of full-wave simulations beyond

the first-order Born approximation, the extension of the evolving scattering network model to multiple scattering events will be the subject of future study.

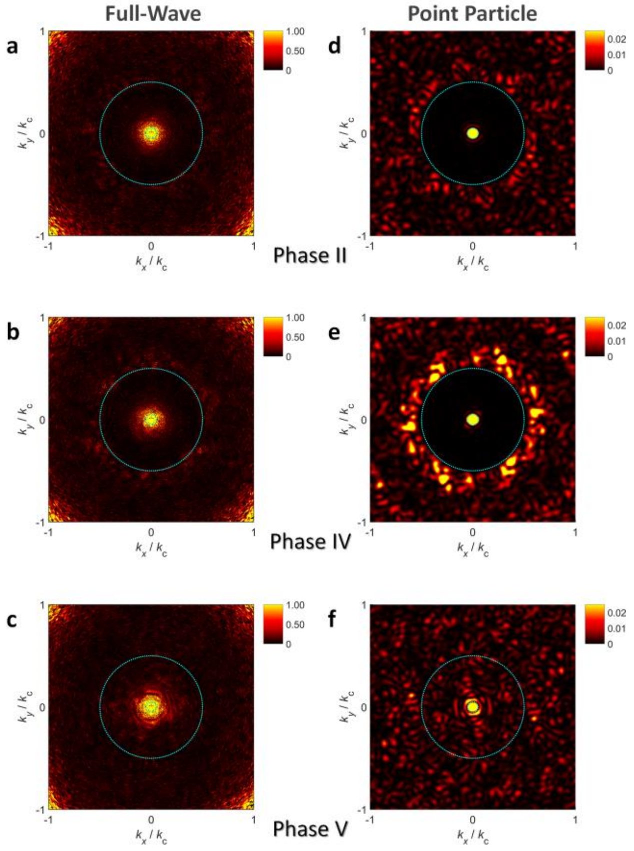

Supplementary Figure 14. Comparison between full-wave simulation and point particle assumption. a-f, Examples of the structure factors $S(\mathbf{k})$ obtained from the analysis based on the **a-c,** full-wave simulations and **d-f,** point particle assumption: **a,d,** phase II, **b,e,** phase IV, and **c,f,** phase V in Fig. 6 in the main text. Each material is composed of 500 particles.

Supplementary Algorithm 1. Pseudo-code for evolution processes. This method describes the pseudo-code for calculating particle positions based on the evolution process. For the results of Fig. 4, the pseudo-code is developed to assume n_{pre} pre-deposited particles and n_{post} post-deposited particles around the evolution process, which are governed by the Poisson process.

Initialize a pseudorandom number generator for the Monte Carlo discretization

Initialize the total number of particles n, which will be deposited inside a material

Initialize the number of existing particles n_{pre} ($\geq$ 1)

Initialize the number of particles included after the evolution process n_{post} ($\geq$ 0)

Initialize the real space of interest $\mathbf{\Omega}$ (2D circle in the results of this work)

Initialize the real space $\mathbf{\Omega}_{\mathrm{evol}}$ for depositing particles during the evolution ($\mathbf{\Omega}_{\mathrm{evol}} \subset \mathbf{\Omega}$)

Initialize the real space $\mathbf{\Omega}_{\mathrm{pre}}$ for pre-deposited particles ($\mathbf{\Omega}_{\mathrm{pre}} \subset \mathbf{\Omega}$)

Initialize the real space $\mathbf{\Omega}_{\mathrm{post}}$ for post-deposited particles ($\mathbf{\Omega}_{\mathrm{post}} \subset \mathbf{\Omega}$)

Initialize the reciprocal space of interest $\mathbf{K}$ for the evolution process ($\mathbf{K_L}$ in Fig. 3-6)

Initialize the reciprocal space of monitoring $\mathbf{K_M}$ ($\mathbf{K_L}$ and $\mathbf{K_S}$ in this work)

Initialize the number of candidate positions n_{MC} for the Monte Carlo discretization

Initialize the preference function $\Pi(w_p^{\mathbf{K}})$

Define a set of the randomly selected n_{MC} positions $\mathbf{\Omega}_{\mathrm{MC}} = \{\mathbf{r}_k | k = 1, 2, \ldots, n_{\mathrm{MC}}, \mathbf{r}_k \in \mathbf{\Omega}\}$

for every n' where $0 < n' \leq n_{\mathrm{pre}}$ **do**

  Set $\mathbf{r}_{n'}$ randomly selected from $\mathbf{\Omega}_{\mathrm{MC}} \cap \mathbf{\Omega}_{\mathrm{pre}}$

  Calculate $\langle S_{n'} \rangle_{\mathbf{K}}$ for $\mathbf{K} = \mathbf{K_M}$ using Eqs (6) and (1) (or Eqs (6) and (5))

end for

for every n' where $n_{pre} < n' \leq n - n_{post}$ **do**

Calculate $\rho_{n'}^{\mathbf{K}}(\mathbf{r})$ for $\mathbf{r} \in \mathbf{\Omega}_{MC} \cap \mathbf{\Omega}_{evol}$ using Eq. (7) and $\Pi(w_p^{\mathbf{K}})$

Select $\mathbf{r}_{min}$ satisfying $\rho_{n'}^{\mathbf{K}}(\mathbf{r}_{min}) = \min[\rho_{n'}^{\mathbf{K}}(\mathbf{r} \in \mathbf{\Omega}_{MC} \cap \mathbf{\Omega}_{evol})]$

Set $\mathbf{r}_{n'} = \mathbf{r}_{min}$

Calculate $<S_{n'}>_{\mathbf{K}}$ for $\mathbf{K} = \mathbf{K}_{\mathbf{M}}$ using Eqs (6) and (1) (or Eqs (6) and (5))

end for

for every n' where $n - n_{post} < n' \leq n$ **do**

Set $\mathbf{r}_{n'}$ randomly selected from of $\mathbf{\Omega}_{MC} \cap \mathbf{\Omega}_{post}$

Calculate $<S_{n'}>_{\mathbf{K}}$ for $\mathbf{K} = \mathbf{K}_{\mathbf{M}}$ using Eqs (6) and (1) (or Eqs (6) and (5))

end for

References

1. Pathria, R. K. & Beale, P. D. *Statistical Mechanics* (Elsevier/Academic Press, 2011).
2. Gonis, A. & Butler, W. H. *Multiple scattering in solids* (Springer Science & Business Media, 1999).
3. Arfken, G. B. & Weber, H. J. *Mathematical methods for physicists* (American Association of Physics Teachers, 1999).
4. Uche, O. U., Torquato, S. & Stillinger, F. H. Collective coordinate control of density distributions. *Phys. Rev. E* **74**, 031104 (2006).
5. Torquato, S. Hyperuniform states of matter. *Phys. Rep.* **745**, 1-95 (2018).
6. Osnabrugge, G., Leedumrongwatthanakun, S. & Vellekoop, I. M. A convergent Born series for solving the inhomogeneous Helmholtz equation in arbitrarily large media. *J. Comput. Phys.* **322**, 113-124 (2016).
